# Supplementary material for: Synthesis and direct assay of large macrocycle diversities by combinatorial late-stage modification at picomole scale
Source: Nat Commun. 2022 Jul 2;13:3823. doi: 10.1038/s41467-022-31428-8 (PMC9250534; doi:10.1038/s41467-022-31428-8)
Supplement: Supplementary file 1 — Supplementary Information [file 41467_2022_31428_MOESM1_ESM.pdf]

Supplementary Information

Synthesis and direct assay of large macrocycle diversities by combinatorial late-stage modification at picomole scale

Sevan Habeshian[1], Manuel Leonardo Merz[1], Gontran Sangouard[1], Ganesh Kumar Mothukuri[1], Mischa Schüttel[1], Zsolt Bognár[1], Cristina Díaz-Perlas[1], Jonathan Vesin[2], Julien Bortoli Chapalay[2], Gerardo Turcatti[2], Laura Cendron[3], Alessandro Angelini[4,5], and Christian Heinis[1]*

[1]Institute of Chemical Sciences and Engineering, Ecole Polytechnique Fédérale de Lausanne (EPFL), CH-1015 Lausanne, Switzerland.

[2]Biomolecular Screening Facility, Ecole Polytechnique Fédérale de Lausanne (EPFL), Lausanne, Switzerland.

[3]Department of Biology, University of Padova, 35131 Padova, Italy.

[4]Department of Molecular Sciences and Nanosystems, Ca' Foscari University of Venice, Via Torino 155, Venezia Mestre, Venice 30172, Italy.

[5]European Centre for Living Technologies (ECLT), Ca' Bottacin, Dorsoduro 3911, Calle Crosera, Venice 30124, Italy.

*Corresponding author. Email: christian.heinis@epfl.ch

Index

Supplementary Discussion

Overall structure of human α-thrombin in complex with M1 4

Overall structure of macrocycle M1 4

Interactions between human α-thrombin and M1 4

Supplementary Tables

Supplementary Table 1. Macrocycle libraries 6

Supplementary Table 2. Statistics on X-ray structure data collection and refinement 7

Supplementary Table 3. Interactions in X-ray structure 9

Supplementary Figures

Supplementary Figure 1. Acylation reactions using DIPEA as base 13

Supplementary Figure 2. Acylation reactions using different bases 14

Supplementary Figure 3. Acylation reactions using DABCO as base 15

Supplementary Figure 4. Acylation of model scaffolds 2 to 5 16

Supplementary Figure 5. Scaffolds containing an N-terminal amino group 18

Supplementary Figure 6. Physicochemical properties of libraries 19

Supplementary Figure 7. Thrombin inhibitors M1 to M5 20

Supplementary Figure 8. Structure of M1 bound to thrombin 21

Supplementary Figure 9. Scaffolds synthesized for Library 2 (MDM2 screen) 22

Supplementary Figure 10. Carboxylic acids 23

Supplementary Figure 11. Repetition of MDM2 screen with selected acids 24

Supplementary Figure 12. HPLC chromatograms of MDM2 binders 25

Supplementary Figure 13. Binding site of M10 and competition with nutlin-3a 26

Supplementary Figure 14. Binding of macrocycles to MDM2 measured by SPR 27

Supplementary References 28

Supplementary Discussion

Overall structure of human α-thrombin in complex with M1

Human α-thrombin consists of two polypeptide chains of 36 (light chain) and 259 amino acid residues (heavy chain) covalently linked via a disulfide bridge (Cys122 of H-chain with Cys1 of L-chain). X-ray structure analysis of crystals formed by α-thrombin (light- and heavy-chain) and macrocycle M1 showed four nearly identical copies of heavy- and light-chains of human α-thrombin in the asymmetric unit. The four light/heavy chains of α-thrombin are named A/B, C/D, E/F, and H/L. The structure of H/L was used for all calculations and for preparing the structure figures. The light-chain of human α-thrombin can be traced unambiguously from Glu1C to Ile14K. The amino terminal residues (Thr1H to Gly1D) and the carboxyl-terminal residues Asp14L (except for light-chain C and E), Gly14M and Arg15 are undefined and not visible in the Fourier map. The electron density of the heavy-chain is clearly visible for all residues with the exception of few amino acids that are part of the surface flexible autolysis loop (Trp148 to Val149C). The carboxyl-terminal residue Glu247 lacks adequate electron density. Minor differences occur at the level of flexible and less defined loops or in the orientation of exposed peripheral side chains. The overall structure of human α-thrombin bound to the macrocycle does not show any striking rearrangements of the main backbone if compared to other human α-thrombin structures, neither in the apo form, nor in complex with inhibitors.

Overall structure of macrocycle M1

The electron density of the macrocycle M1 is well-defined allowing an unambiguous assignment of group orientations for all the four protein complexes present in the asymmetric unit. The numbering of the atoms in M1 is shown in Supplementary Fig. 8b. No classical secondary structure elements and no non-covalent intra-molecular interactions are found in the macrocycle. The molecule appears to adopt a chair-like conformation that fits well the shape of the catalytic pocket.

Interactions between human α-thrombin and M1

The M1 macrocycle fits well into the cleft formed by the active site and the surrounding substrate pockets covering a protein surface of 400.5 $Å^2$ as calculated by a software tool[1]. The macrocycles'

conformations and interactions are equivalent in the four active sites of the four-thrombin molecules present in the asymmetric unit. A large portion of interactions of M1 with human α-thrombin are mediated by the 5-chlorothiophene-2-carboxamide functional group that accommodates in the primary specificity S1 pocket. This group is trapped in the pocket by a hydrogen bond with the main chain of Gly219 (M1 N7 with Gly219 O) and a molecule of H_2O that bridges the oxygen O9 of M1 with the main chain nitrogen of Gly193 N (Gly193 N, Supplementary Table 3) and the main chain nitrogen of Ser195 (Ser195 N, Supplementary Table 3). 5-chlorothiophene-2-carboxamide is further involved in a network of polar contacts with the main chain of the nearby Cys191 (M1 O9 with Cys191 O), Glu192 (M1 O9 with Glu192 N), Gly216 (M1 N7 with Gly216 O and M1 S15 with Gly216 N), Trp215 (M1 S15 with Trp215 N) and the side chain of Cys220 (M1 N7 with Cys220 S; Supplementary Table 3). The chlorine atom 5-chlorothiophene-2-carboxamide functional group points toward the bottom of the S1 pocket where it forms likely a halogen-aromatic π interaction (4.0 Å) with the aromatic ring of Tyr228 (Fig. 3f). The main chain nitrogen N4 and oxygen O17 of M1 form hydrogen bonds with the main chain oxygen of Gly216 (Gly216 O) and nitrogen of Gly216 (Gly216 N), respectively (Supplementary Table 3). Additionally, the main chain nitrogen N4 and oxygen O17 of M1 can form polar contacts with the main chain nitrogen of Gly219 (Gly219 N) and oxygen of Gly216 (Gly216 O), respectively (Supplementary Table 3). Similarly, the main chain nitrogen N18 of M1 can form two polar contacts with the side chain carboxylic group of Glu192 (Glu192 OE1 and OE2; Supplementary Table 3). Finally, a molecule of H_2O bridges the main chain nitrogen N27 of M1 with the main chain oxygen of Glu97A (Glu97A O, Supplementary Table 3). Importantly, the binding of M1 to human α-thrombin is mediated by multiple hydrophobic contacts by main and side chains of adjacent enzyme residues (Supplementary Table 3). The macrocycle backbone (C20-C24), including the disulfide bridge S21-S22, lays towards the hydrophobic cage shaped by the side chains of residues His57, Tyr60A, Trp60D (proximal S2 pocket) and Leu99 (distal S3 pocket). The valine side chain (C28-C31) bends the other side of the ring toward the hydrophobic pocket formed by Ile174 and Trp215. Finally, the C35 – C40 phenyl ring run on top of a thrombin loop (Gly216 – Cys220).

Supplementary Tables

Supplementary Table 1. Macrocycle libraries. Size, diversity and physicochemical properties of the macrocycle libraries. *) Backbones: different macrocyclic skeletons, ignoring peripheral groups; backbones having the same atoms but different conformational constrains imposed by peripheral groups (e.g. N-methylation, cyclic side chain, etc.) or bonds are considered as different backbones.

	**Library 1**	**Library 2**
**Library size**		
Number macrocycles	4,608	19,968
**Structural diversity**		
Number scaffolds	384	192
Different backbones*	144	96
**Chemical diversity**		
Total number of building blocks	41	116
Different amino acids	27	10
Different carboxylic acids	12	104
Flanking thiol groups	2	2
**Physicochemical properties**		
Molecular weight (average)	624	684
cLogP (average)	0.8	2.1
Polar surface area (average)	228 Å^2	211 Å^2
Number H-bond donors (average)	5.23	4.31

Supplementary Table 2. Statistics on X-ray structure data collection and refinement (PDB 6Z48). A single crystal was used to collect all diffraction data. Highest-resolution shell statistics are shown within brackets.

**Data collection**	
Wavelength (Å)	0.9795
Space group	$P2_1$
Cell parameters	
a, *b*, *c* (Å); α, β, γ (°)	56.25, 100.57, 108.90; 90, 90.11, 90
Resolution (Å)	73.88 – 2.27 (2.35 – 2.27)
Observations	104,136 (8,954)
Unique	52,645 (4,712)
Multiplicity	2.0 (1.9)
R_{merge}	0.0411 (0.2527)
R_{pim}	0.021 (0.210)
$\langle I / \sigma(I) \rangle$	16.69 (3.35)
$CC_{1/2}$	0.998 (0.794)
Completeness (%)	93.86 (84.19)
Wilson B-factor	24.25
**Refinement**	
No. reflections (Used for R_{free} calculation)	52,631 (4,712)
R_{work} / R_{free}	0.189 / 0.242
Number non-hydrogen atoms	9,784
protein (chains A, B, C, D, E, F, H, L)	9,221
ligands (M1, Na^+)	164
solvent	399

**Geometry**	
RMSD values	
bond lengths (Å)	0.013
bond angles (°)	1.90
Ramachandran plot (%)	
most favored	95.00
additionally allowed	4.91
outliers	0.09
Average B-factor	28.08

Supplementary Table 3. Atoms of the macrocycle of M1 forming interactions with atoms and residues of human α-thrombin (chymotrypsin numbering). Interactions have distances shorter than 4.0 Å and were defined using the software LIGPLOT+ by the web server PROFUNC.

Thrombin atom / residue	M1 atom	Distance (Å)	Interaction
O / Cys191	O9	3.58	PI
N / Glu192	O9	3.61	PI
OE1 / Glu192	N18	3.66	PI
OE2 / Glu192	N18	3.35	PI
N / Trp215	S15	3.73	PI
N / Gly216	S15	3.59	PI
N / Gly216	O17	3.25	HB
O / Gly216	N4	2.92	HB
O / Gly216	N7	3.84	PI
O / Gly216	O17	3.35	PI
N / Gly219	N4	3.89	PI
O / Gly219	N7	3.17	HB
S / Cys220	N7	3.82	PI
N / Gly193 (H_2O)	O9	2.85 (2.80)	HB
N / Ser195 (H_2O)	O9	2.85 (3.17)	HB
O / Glu97A (H_2O)	N27	3.20 (2.80)	HB

Thrombin atom / residue	M1 atom	Distance (Å)
CD2 / His57	S21	3.84
CE1 / Tyr60A	C24	3.74

CE2 / Tyr60A	C23	3.62
CE2 / Tyr60A	C24	3.86
CZ / Tyr60A	C23	3.65
CZ / Tyr60A	C24	3.34
OH / Tyr60A	C23	3.74
OH / Tyr60A	C24	3.05
CZ2 / Trp60D	C23	3.39
CZ3 / Trp60D	S21	3.90
CH2 / Trp60D	S21	3.84
CH2 / Trp60D	C23	3.35
C / Asn98	C30	3.85
O / Asn98	C30	3.62
CD1 / Leu99	C25	3.85
CG1 / Ile174	C31	3.69
CD1 / Ile174	C31	3.65
CG / Asp189	C12	3.73
OD1 / Asp189	C12	3.28
OD2 / Asp189	C12	3.58
C / Ala190	C11	3.88
O / Ala190	C11	3.51
O / Ala190	C12	3.76
CB / Ala190	C13	3.89
C / Cys191	C8	3.85
C / Cys191	O9	3.48
CA / Glu192	O9	3.73
CD / Glu192	N18	3.82
OE1 / Glu192	C5	3.50

OE1 / Glu192	C6	3.70
OG / Ser195	C19	3.28
CG1 / Val213	CL14	3.67
CG1 / Val213	S15	3.55
O / Ser214	C19	3.31
O / Ser214	C20	3.55
CA / Trp215	S15	3.54
CA / Trp215	O17	3.43
C / Trp215	C13	3.49
C / Trp215	S15	3.47
C / Trp215	O17	3.86
O / Trp215	C13	3.48
O / Trp215	CL14	3.60
CB / Trp215	O17	3.45
CG / Trp215	C30	3.56
CD1 / Trp215	C30	3.45
CD2 / Trp215	C30	3.27
NE1 / Trp215	C30	3.20
CE2 / Trp215	C30	3.05
CE3 / Trp215	N34	3.33
CZ2 / Trp215	C30	3.59
CZ3 / Trp215	N34	3.53
N / Gly216	C10	3.46
N / Gly216	C11	3.58
N / Gly216	C12	3.73
N / Gly216	C13	3.66
CA / Gly216	C11	3.76

CA / Gly216	C12	3.81
O / Gly216	C1	3.41
O / Gly216	C2	3.58
O / Gly216	C36	3.83
O / Gly216	C40	3.19
CA / Glu217	C36	3.79
CA / Glu217	C37	3.80
N / Gly219	C1	3.46
N / Gly219	C2	3.79
N / Gly219	C38	3.86
N / Gly219	C39	3.33
O / Gly219	C11	3.14
CA / Gly226	C12	3.86
CA / Gly226	CL14	3.47
C / Gly226	CL14	3.74
N / Phe227	CL14	3.50
O / Phe227	CL14	3.42
CE1 / Tyr228	CL14	3.73
CZ / Tyr228	CL14	3.60
OH / Tyr228	CL14	3.74

Supplementary Figures

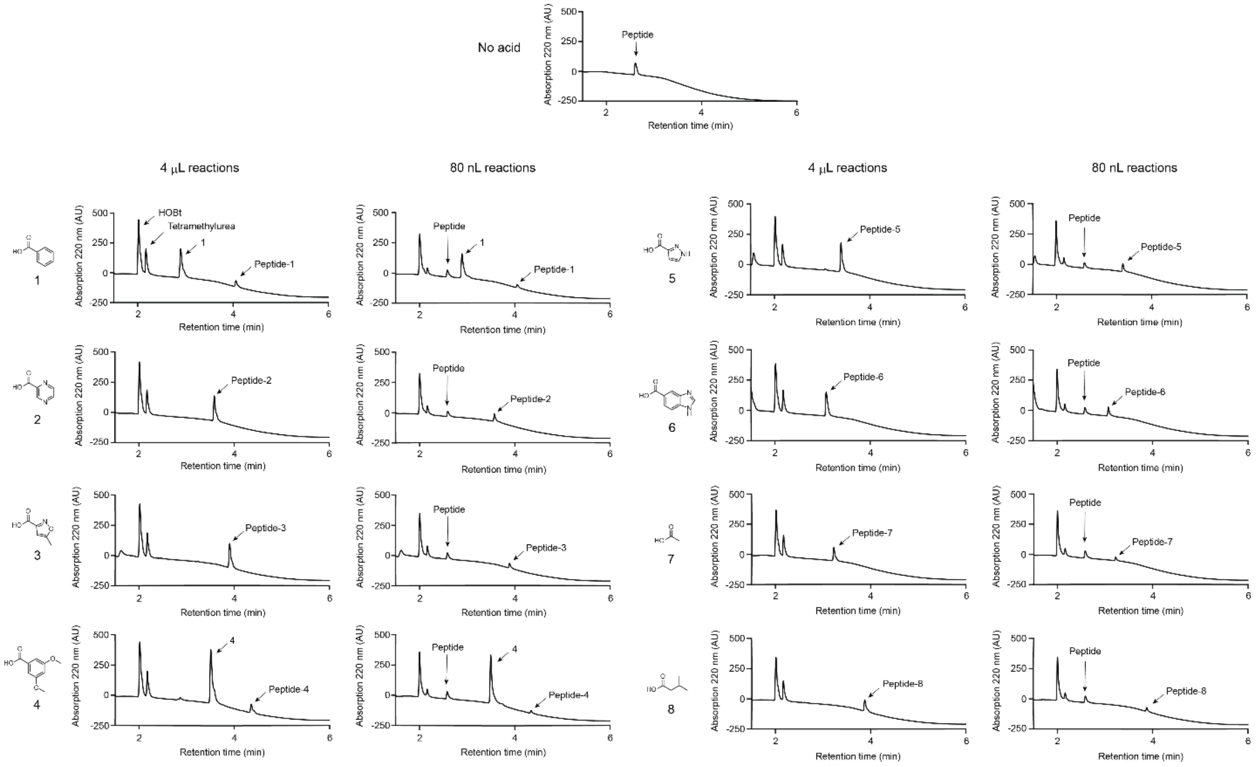

Supplementary Figure 1. Comparison of acylation reactions by pipetting (4 µL volume) and acoustic transfer (80 nL volume) using DIPEA as base. The reactions were diluted 100-fold with water and samples of 5 µL analyzed by LC-MS using a RP column and a 0-60% MeCN/H_2O gradient over 5 minutes. In the case of the 80 nL reaction volume, two samples were pooled.

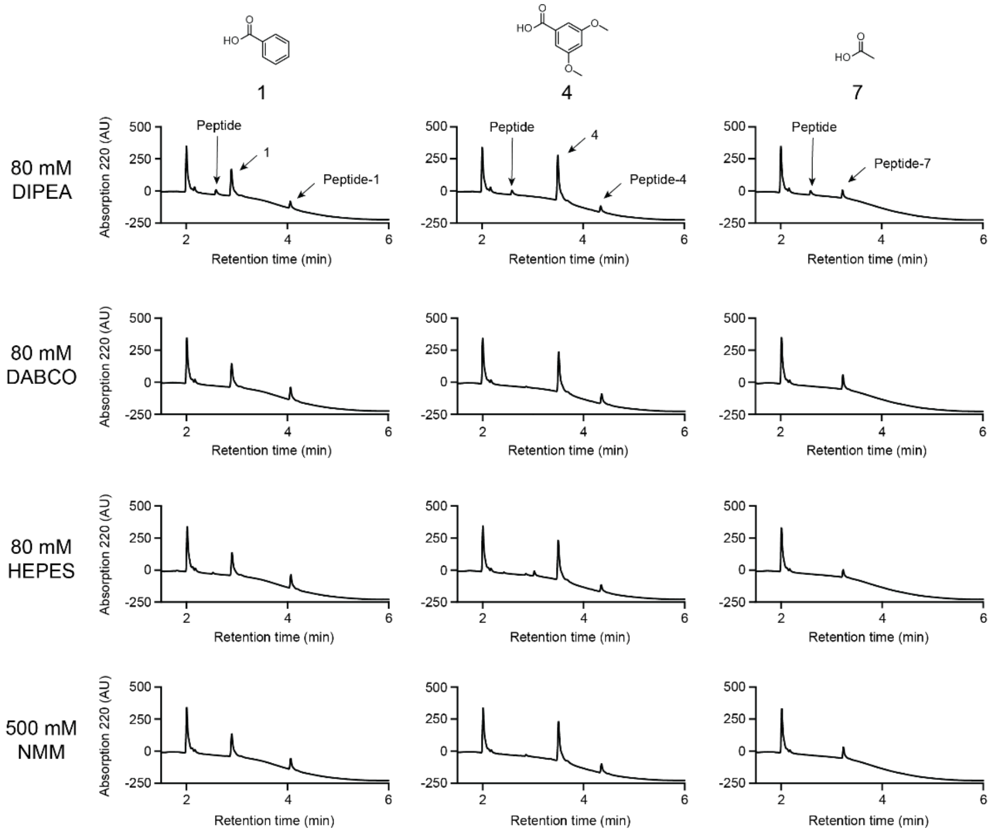

Supplementary Figure 2. Acylation reactions in 80 nL volumes using acoustic dispensing and different bases. Two non-volatile bases (DABCO and HEPES sodium salt) at 80 mM concentration, and a volatile base (NMM) at 500 mM concentration were tested as alternatives to 80 mM DIPEA for the acylation of a model scaffold by three carboxylic acids. The reactions were diluted 100-fold with water and samples of 5 μL analyzed (two pooled reactions) by LC-MS using a RP column and a 0-60% MeCN/H_2O gradient over 5 minutes. While reactions with DIPEA did not go to completion, all other bases resulted in quantitative conversion to product.

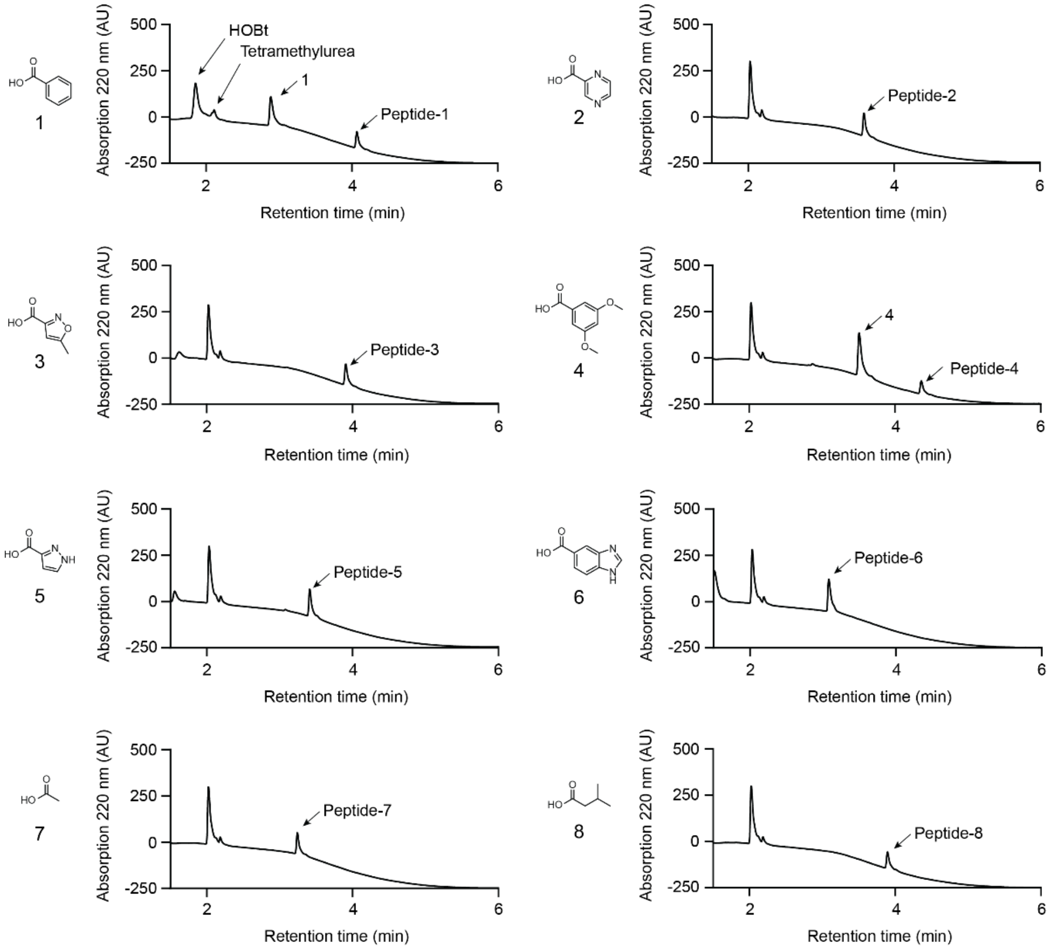

Supplementary Figure 3. Comparison of acylation reactions in 80 nL volumes using acoustic dispending and DABCO as base. The model scaffold 1 was reacted with carboxylic acids **1 – 8** in 80 nL volumes using DABCO as base (80 mM). The reactions were diluted 100-fold with water and samples of 5 μL analyzed (two pooled reactions) were analyzed by LC-MS using a RP column and a 0-60% MeCN/H_2O gradient over 5 minutes.

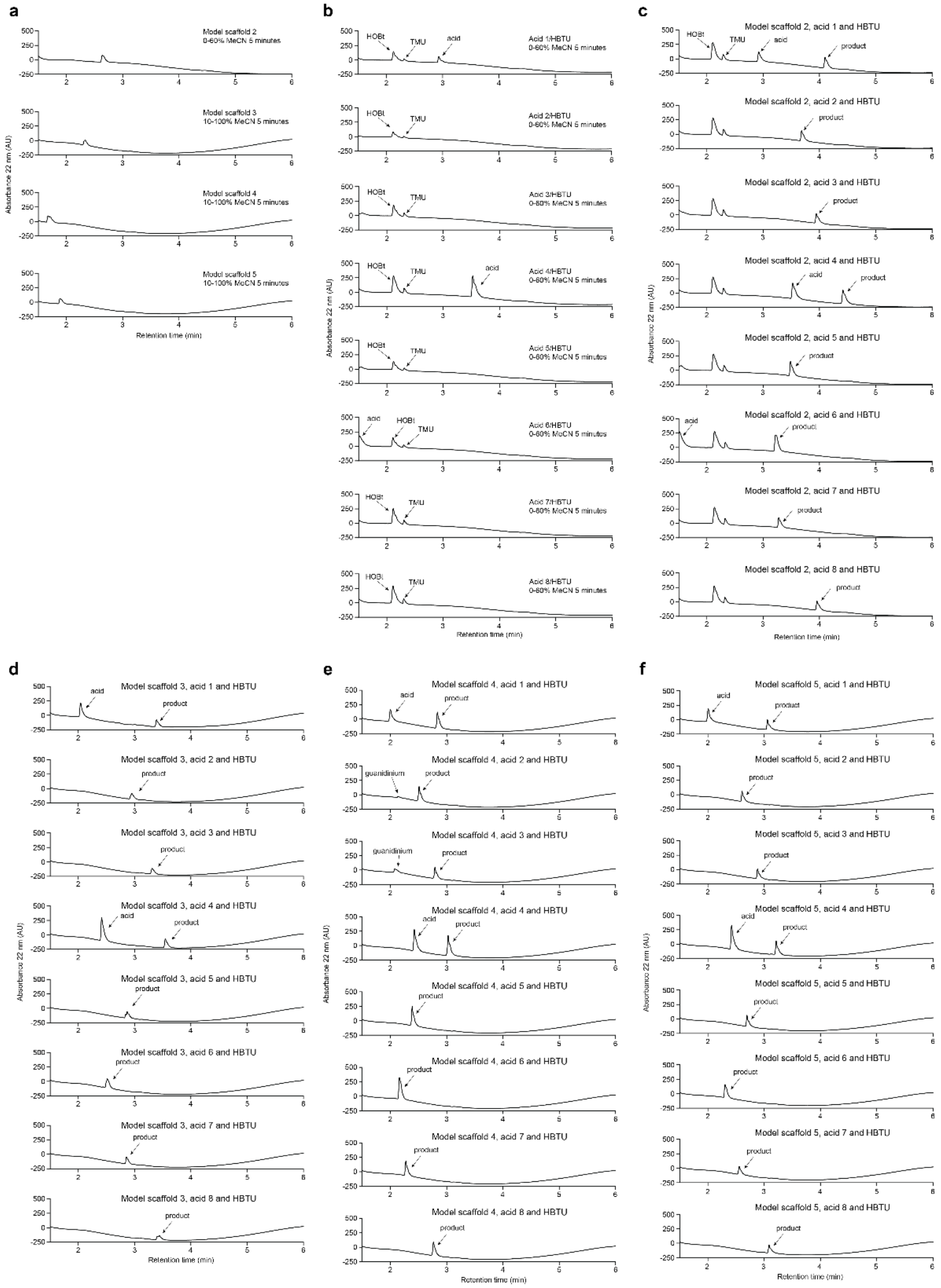

Supplementary Figure 4. Acylation of model scaffolds 2 to 5 in 80 nL volumes and acoustic dispensing. a, LC-MS analysis of model scaffolds. The reactions were diluted 100-fold with water

and samples of 5 μL were analyzed. Solvent B gradient: 0-60% MeCN, 5 minutes for model scaffold 2; 10-100% MeCN, 5 minutes for model scaffolds 3-5. **b**, LC-MS analysis of acids. The reactions were diluted 100-fold with water and samples of 5 μL were analyzed. Solvent B gradient: 0-60% MeCN, 5 minutes. TMU = tetramethyl urea. **c-f,** UHPLC analysis of acylation reactions for model scaffolds 2 (**c**), 3 (**d**), 4 (**e**) and 5 (**f**). The reactions were diluted 100-fold with water and samples of 5 μL were analyzed. Solvent B gradient: 0-60% MeCN, 5 minutes for model scaffold 2; 10-100% MeCN, 5 minutes for model scaffolds 3-5.

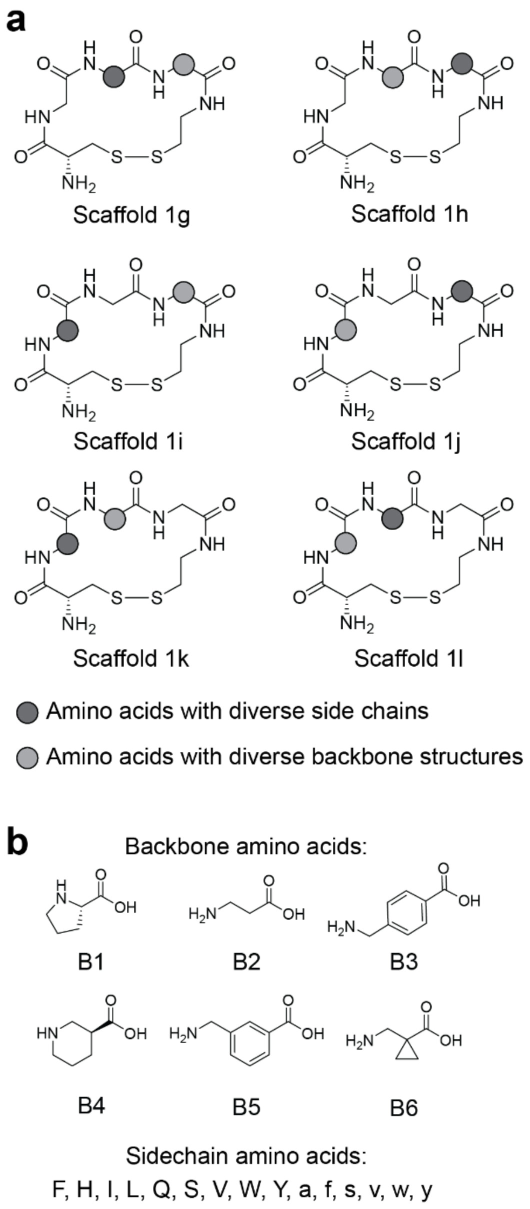

Supplementary Figure 5. Preparation of cyclic peptide scaffolds containing an N-terminal amino group. a, Six different scaffold formats. **b,** Amino acids used for scaffold synthesis.

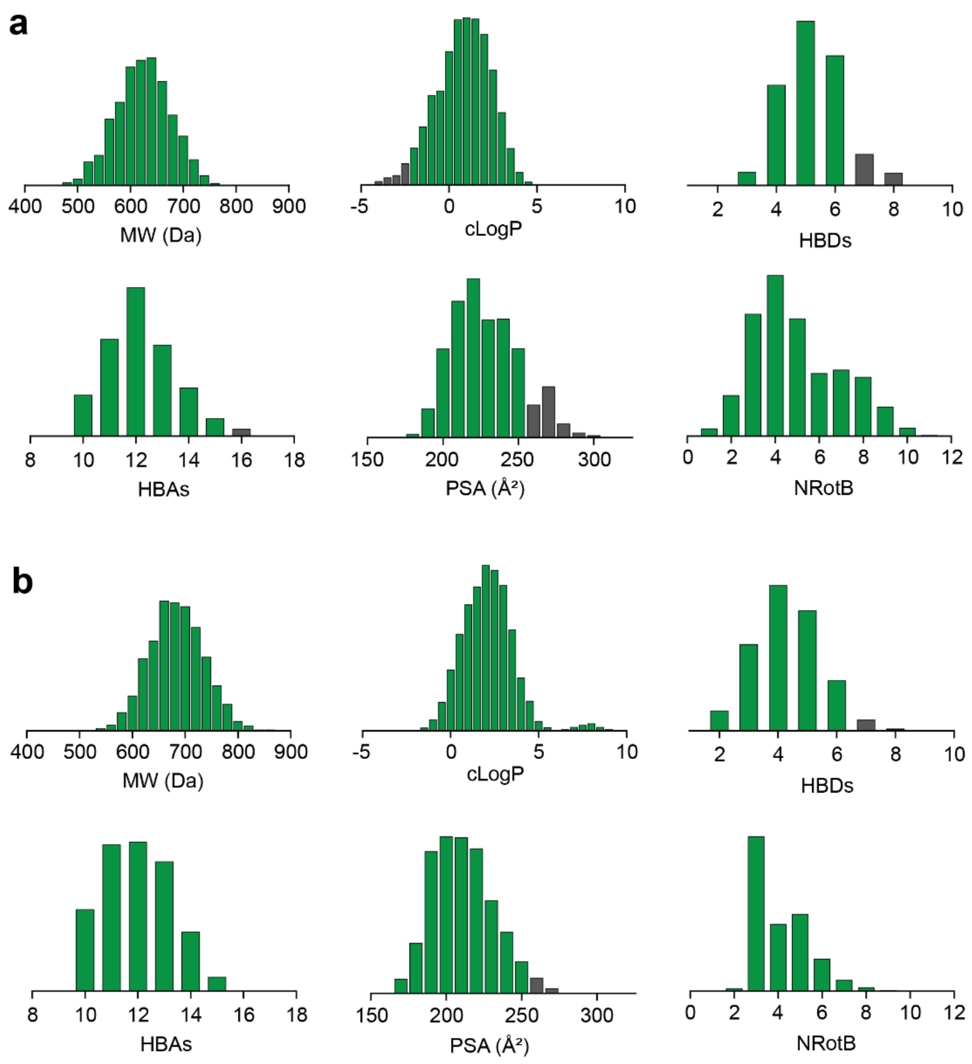

Supplementary Figure 6. Physicochemical properties of Library 1 (4,608 compounds; thrombin screen) and Library 2 (19,968 compounds; MDM2 screen). Properties were calculated for all the compounds in each library using DataWarrior software. Regions compliant with Kihlberg's rules for permeability[2,3] are colored green. The majority of both libraries fall in a space that is predicted to be cell permeable. MW = molecular weight, cLogP = calculated n-octanol/water partition coefficient, HBD = hydrogen bond donors, HBA = hydrogen bond acceptors, PSA = polar surface area, NRotB = number of rotatable bonds. **a,** Library 1 (for thrombin screen). **b,** Library 2 (for MDM2 screen).

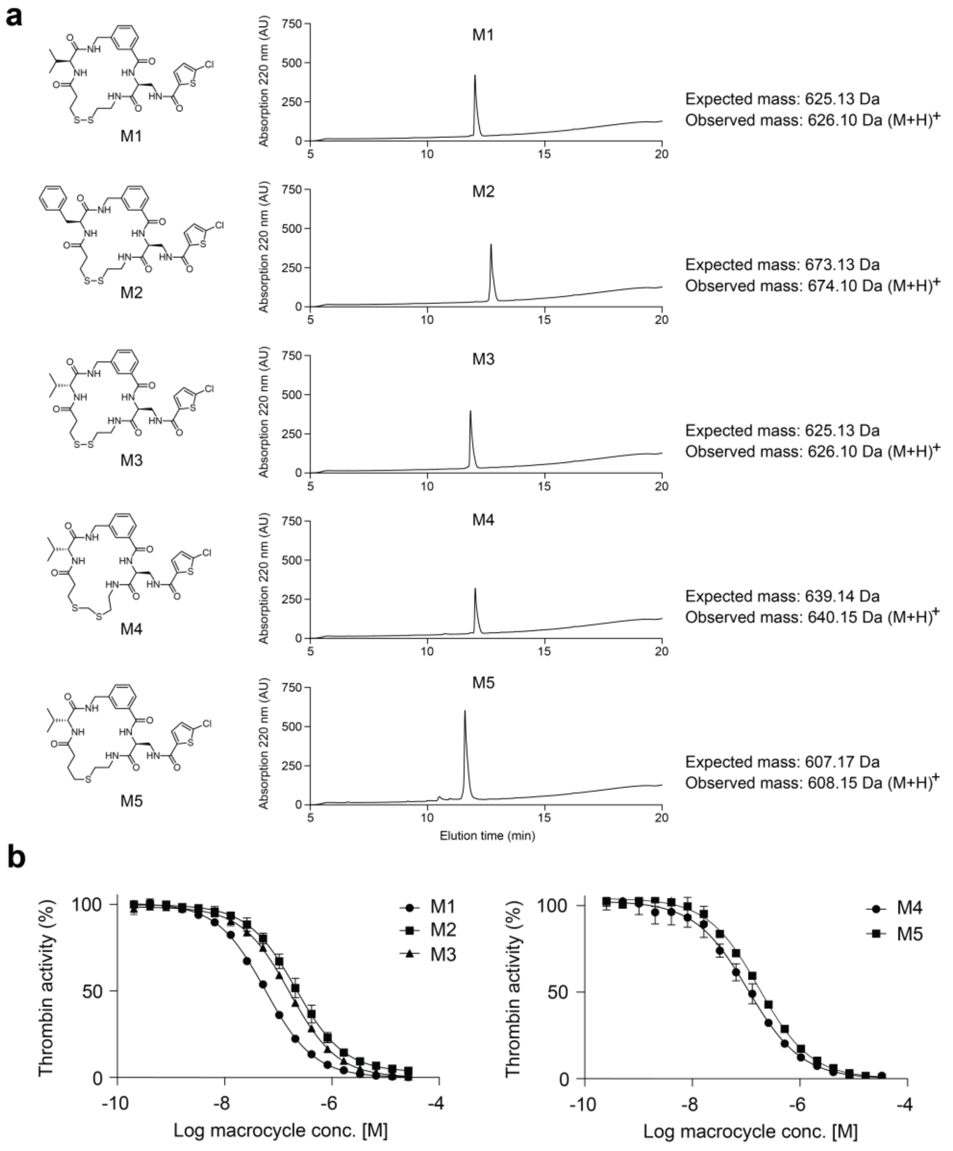

Supplementary Figure 7. Thrombin inhibitors M1 to M5. a, Chemical structures and analytical HPLC chromatograms obtained using a 0-100% MeCN/H_2O gradient over 15 minutes. **b,** For all macrocycles, an 18-point, two-fold serial dilution was performed in 50 µL volumes. Thrombin was added (50 µL, 2 nM final conc.), followed 10 minutes later by fluorogenic substrate Z-Gly-Gly-Arg-AMC (50 µL, 50 µM final conc.). The increase in fluorescence was measured over 30 minutes. Residual thrombin activity was determined by dividing the slope of fluorescence intensity over time for each well by the slope of control wells without macrocycle. Mean values and SDs are indicated for three independent thrombin inhibition measurements.

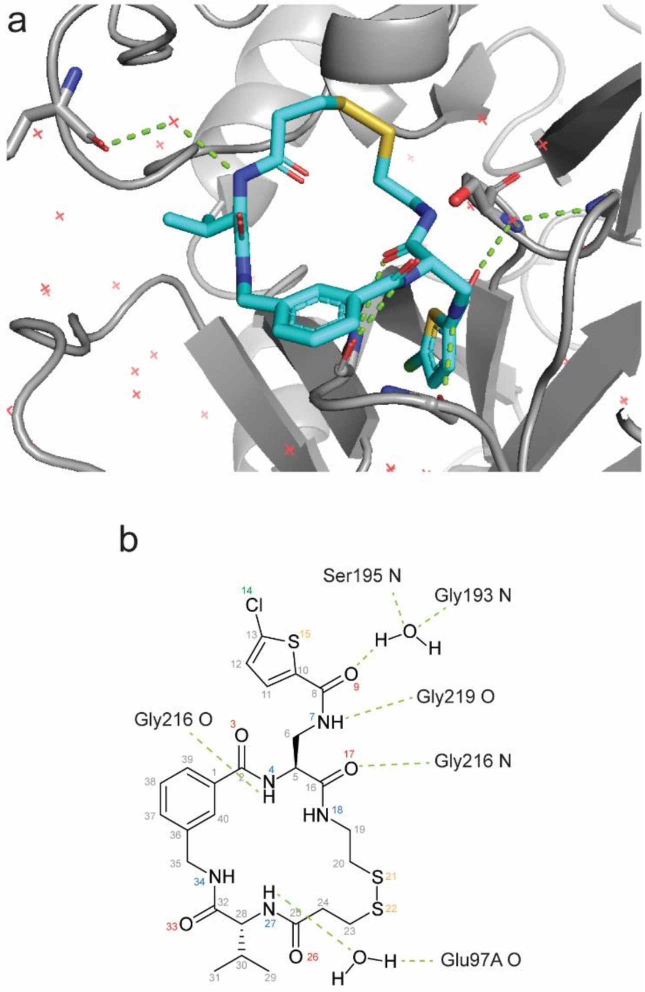

Supplementary Figure 8. Structure of M1 bound to thrombin. a, X-ray structure of M1 bound to thrombin. M1 is zoomed in and the H-bond interactions are indicated. **b,** Chemical structure of M1 and H-bond interactions formed with thrombin.

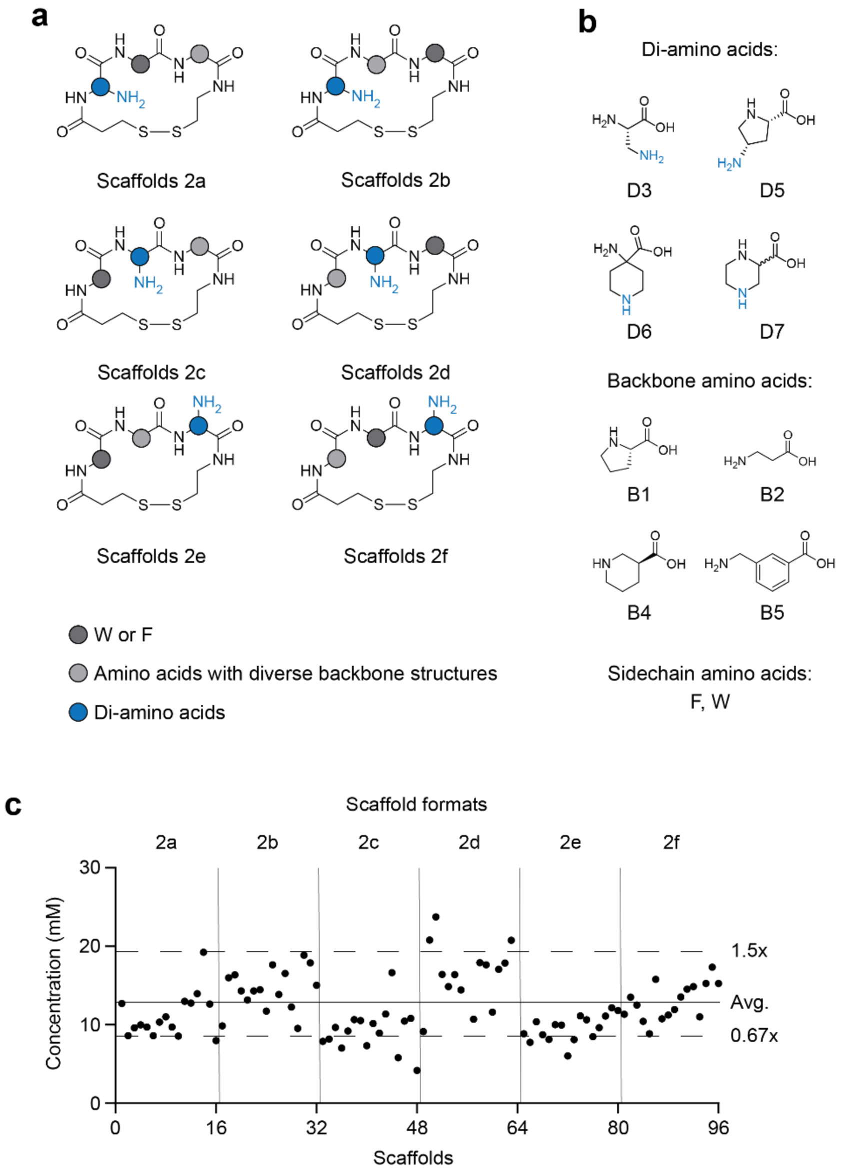

Supplementary Figure 9. Scaffolds synthesized for Library 2 (MDM2 screen). a, Format of scaffolds in Library 2. **b,** Amino acids used for the scaffold library synthesis. All combinations of four di-amino acids, four backbone amino acids, two sidechain amino acids, and six sub-library formats, were synthesized. **c,** Yields of tryptophan-containing scaffolds after cyclative release. The average concentration of scaffold was 12.9 mM as determined by nanodrop absorbance. Two of the scaffolds had concentrations higher than 30 mM and these datapoints are thus not shown in the graph. The average purity measured by LC-MS was around 90%.

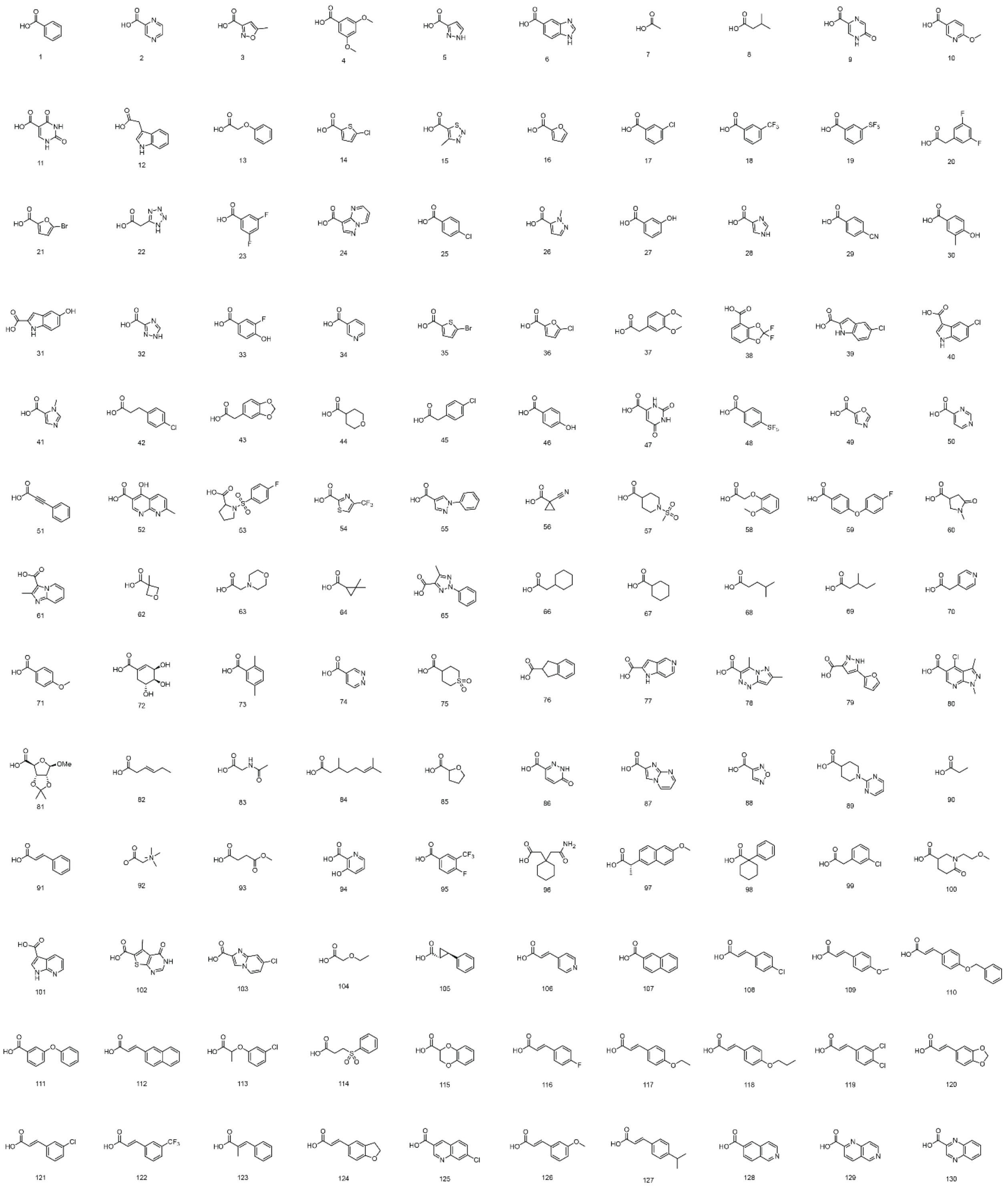

Supplementary Figure 10. Carboxylic acids. Overview of carboxylic acids used to acylate peripheral amines in cyclic peptide scaffolds.

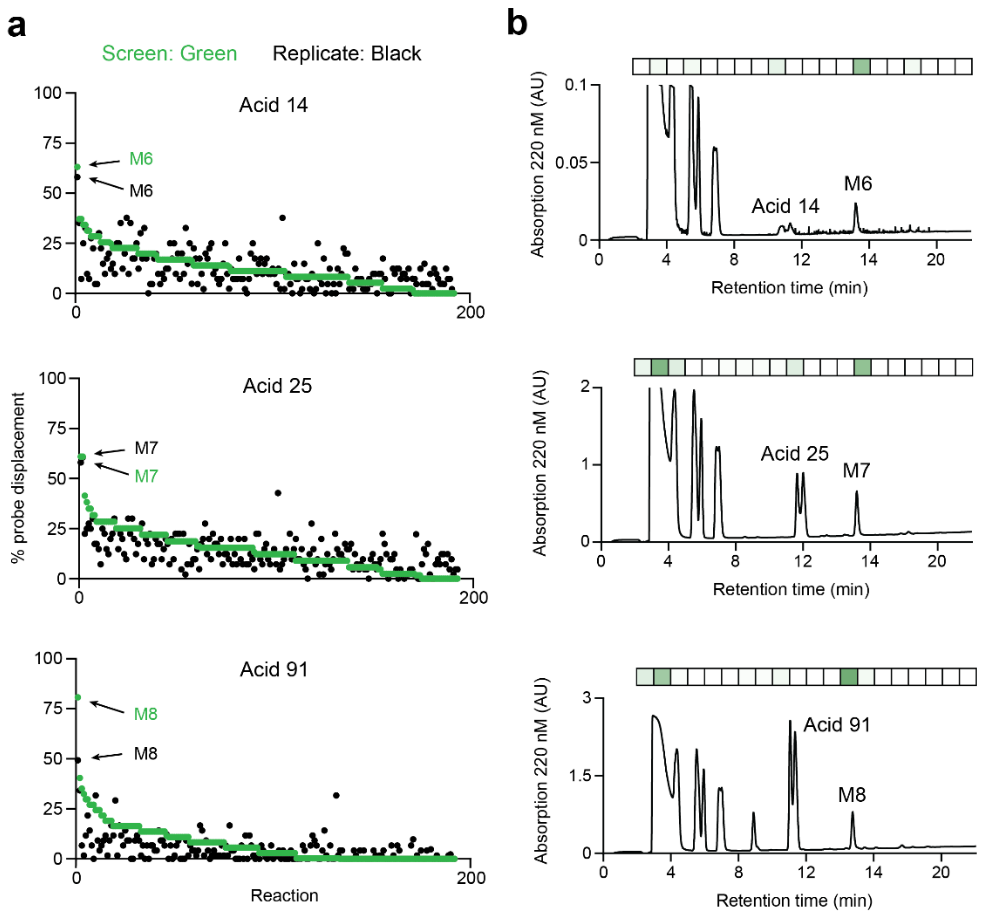

Supplementary Figure 11. Repetition of MDM2 screen with acids that showed strong reporter peptide displacement in screen, and identification of active species in reactions of M6 to M8. a, Data of initial screen is shown in green and data of replicate is shown in black. For each of the two independent experiments, the reactions were performed once and the thrombin inhibition was measured once. Macrocycles are ordered according to their activity measured in the initial screen. **b,** Chromatographic separation acylation reaction yielding M6, M7 and M8 and analysis of fractions for MDM2-binding species.

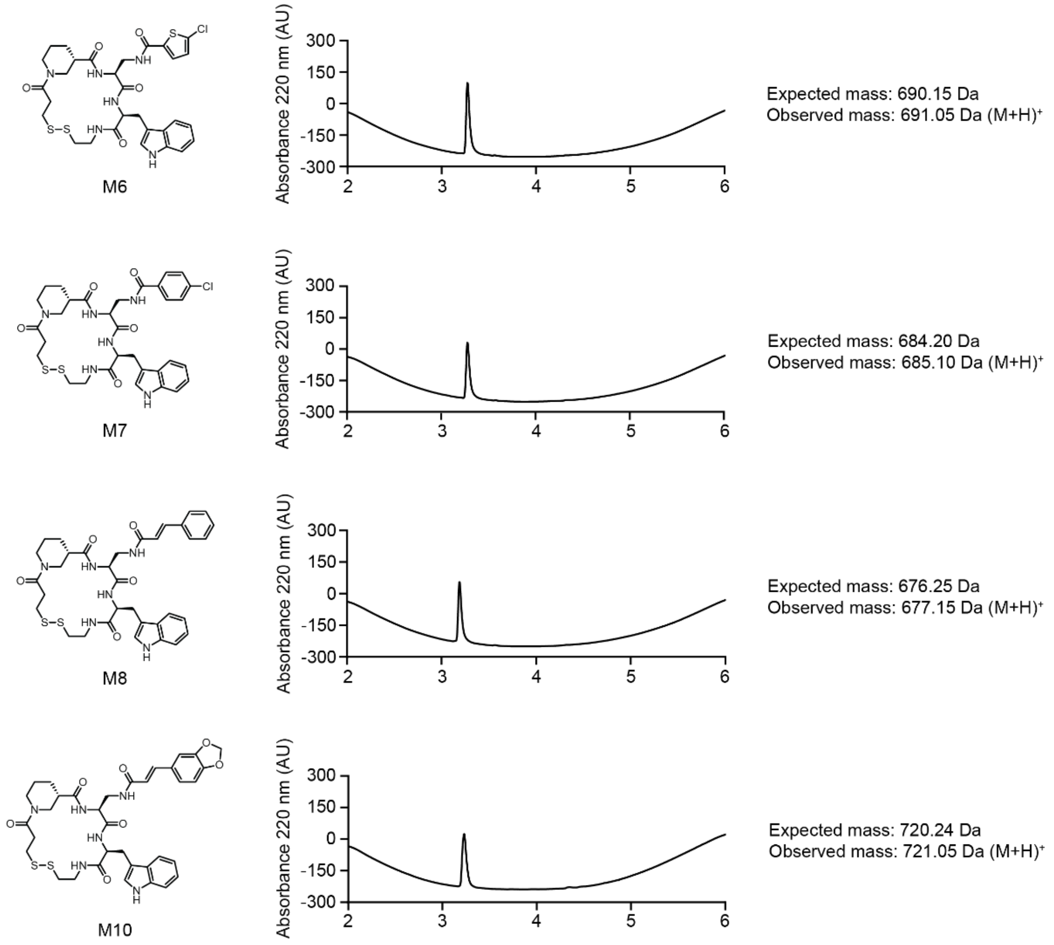

Supplementary Figure 12. Macrocycles identified in screen against MDM2. Chemical structures and analytical HPLC chromatograms for macrocycles, that were synthesized and HPLC-purified, are shown. The expected and experimentally observed masses are indicated.

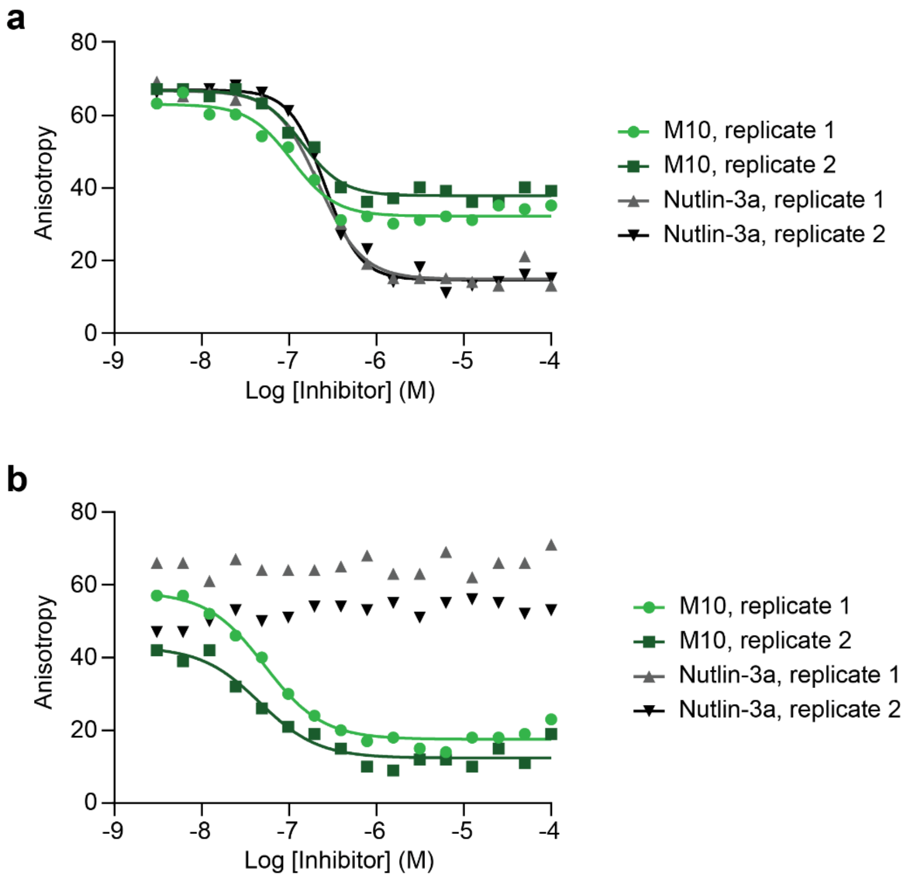

Supplementary Figure 13. Studying the binding site of M10 by competition binding experiments. a, Displacement of the FP53 linear peptide probe by M10 and nutlin-3a measured by fluorescence polarization. The displacement was measured in two independent experiments and the two data sets are displayed individually as replicate 1 and replicate 2. **b,** Displacement of the F-M10 macrocycle probe by M10 and nutlin-3a. The displacement was measured in two independent experiments and the two data sets are displayed individually as replicate 1 and replicate 2.

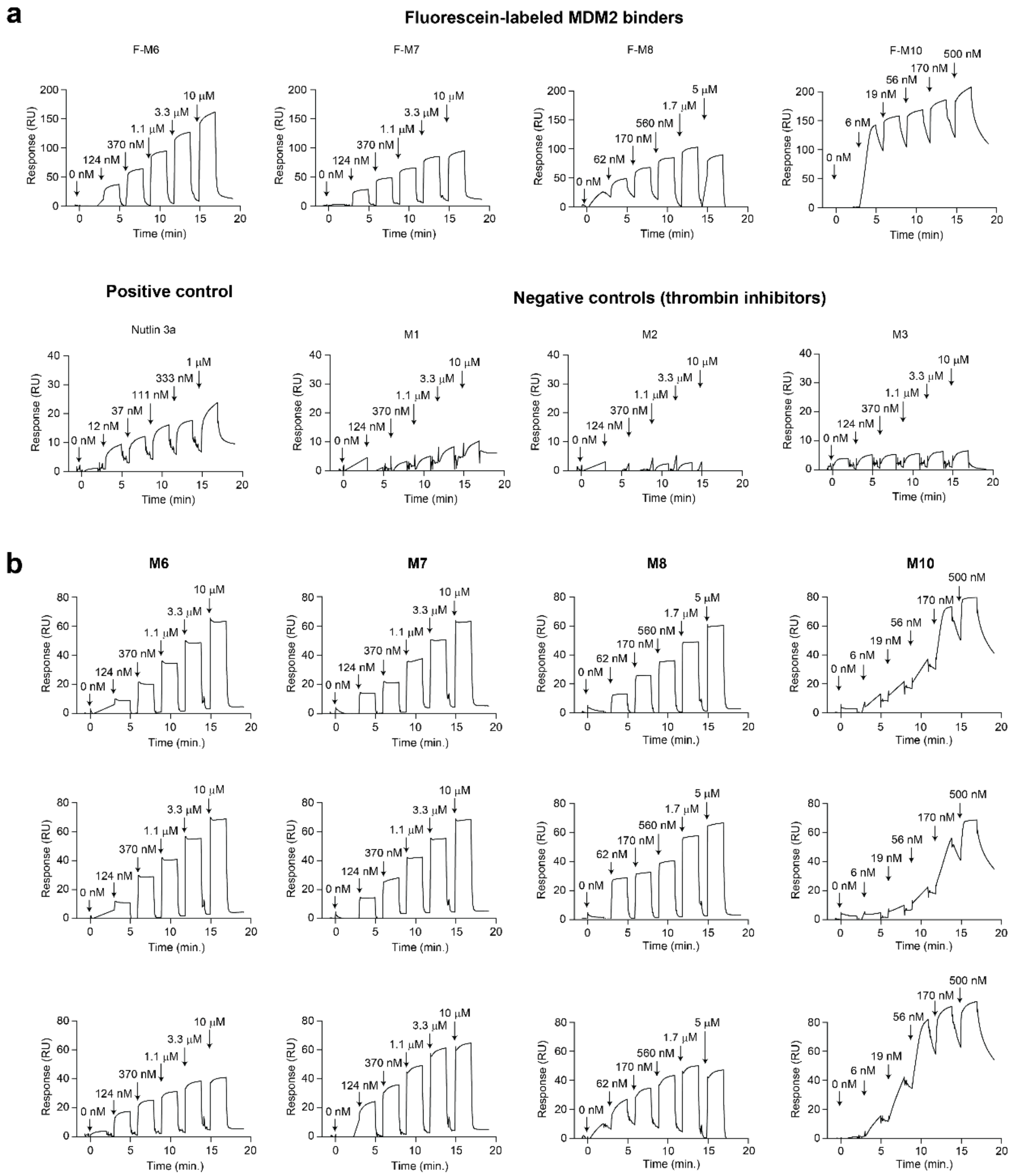

Supplementary Figure 14. Binding of macrocycles to MDM2 measured by SPR. a, Single-cycle SPR sensorgrams for fluorescein-labeled macrocycles, positive control nutlin-3a (not fluorescein labeled), and negative controls (thrombin inhibitors; not fluorescein labeled). RU, response unit. **b,** Single-cycle SPR sensorgrams for macrocycles M6, M7, M8 and M10 (not fluorescein-labeled), performed in triplicate.

Supplementary References

1. Voss, N. R. & Gerstein, M. 3V: Cavity, channel and cleft volume calculator and extractor. *Nucleic Acids Res.* **38**, W555–W562 (2010).
2. Matsson, P., Doak, B. C., Over, B. & Kihlberg, J. Cell permeability beyond the rule of 5. *Adv. Drug Deliv. Rev.* **101**, 42–61 (2016).
3. Doak, B. C., Over, B., Giordanetto, F. & Kihlberg, J. Oral Druggable Space beyond the Rule of 5: Insights from Drugs and Clinical Candidates. *Chem. Biol.* **21**, 1115–1142 (2014).
