# Supplementary material for: Synthesis and direct assay of large macrocycle diversities by combinatorial late-stage modification at picomole scale
Source: Nat Commun. 2022 Jul 2;13:3823. doi: 10.1038/s41467-022-31428-8 (PMC9250534; doi:10.1038/s41467-022-31428-8)
Supplement: Supplementary file 3 — Reporting Summary [file 41467_2022_31428_MOESM3_ESM.pdf]

nature research

Corresponding author(s): Christian Heinis

Last updated by author(s): May 27, 2022

Reporting Summary

Nature Research wishes to improve the reproducibility of the work that we publish. This form provides structure for consistency and transparency in reporting. For further information on Nature Research policies, see our Editorial Policies and the Editorial Policy Checklist.

Statistics

For all statistical analyses, confirm that the following items are present in the figure legend, table legend, main text, or Methods section.

n/a	Confirmed	
☐	☒	The exact sample size (n) for each experimental group/condition, given as a discrete number and unit of measurement
☐	☒	A statement on whether measurements were taken from distinct samples or whether the same sample was measured repeatedly
☒	☐	The statistical test(s) used AND whether they are one- or two-sided<br>*Only common tests should be described solely by name; describe more complex techniques in the Methods section.*
☒	☐	A description of all covariates tested
☒	☐	A description of any assumptions or corrections, such as tests of normality and adjustment for multiple comparisons
☐	☒	A full description of the statistical parameters including central tendency (e.g. means) or other basic estimates (e.g. regression coefficient) AND variation (e.g. standard deviation) or associated estimates of uncertainty (e.g. confidence intervals)
☒	☐	For null hypothesis testing, the test statistic (e.g. F, t, r) with confidence intervals, effect sizes, degrees of freedom and P value noted<br>*Give P values as exact values whenever suitable.*
☒	☐	For Bayesian analysis, information on the choice of priors and Markov chain Monte Carlo settings
☒	☐	For hierarchical and complex designs, identification of the appropriate level for tests and full reporting of outcomes
☒	☐	Estimates of effect sizes (e.g. Cohen's d, Pearson's r), indicating how they were calculated

Our web collection on statistics for biologists contains articles on many of the points above.

Software and code

Policy information about availability of computer code

Data collection	LC-MS Shimadzu 2020 instrument software, Biacore 8K Control Software, Tecan Infinite M200 Pro instrument software.
Data analysis	Excel (version 2016), Prism (version 5), Pymol (version 2.3.3), CCP4i2 crystallographic package, PHASER, Refmac5, PHENIX, COOT, PISA, PROFUNC, LIGPLOT+, LC-MS Shimadzu 2020 instrument software, Biacore 8K Evaluation Software, Tecan Infinite M200 Pro instrument software, DataWarrier (version 5.2.1).

For manuscripts utilizing custom algorithms or software that are central to the research but not yet described in published literature, software must be made available to editors and reviewers. We strongly encourage code deposition in a community repository (e.g. GitHub). See the Nature Research guidelines for submitting code & software for further information.

Data

Policy information about availability of data

All manuscripts must include a data availability statement. This statement should provide the following information, where applicable:

- Accession codes, unique identifiers, or web links for publicly available datasets
- A list of figures that have associated raw data
- A description of any restrictions on data availability

Supplementary results (X-ray structure and discussion), 3 supplementary tables, and 14 supplementary figures are provided in the Supplementary Information. Raw data is provided in a Source Data file. The atomic coordinates of macrocycle M1 bound to thrombin is deposited in the PDB (https://www.rcsb.org) under the accession code 6Z48 (https://www.rcsb.org/structure/6Z48).

Field-specific reporting

Please select the one below that is the best fit for your research. If you are not sure, read the appropriate sections before making your selection.

☒ Life sciences ☐ Behavioural & social sciences ☐ Ecological, evolutionary & environmental sciences

For a reference copy of the document with all sections, see nature.com/documents/nr-reporting-summary-flat.pdf

Life sciences study design

All studies must disclose on these points even when the disclosure is negative.

Sample size	Determination of yields for scaffolds of Library 1: 45 scaffolds (out of 384 scaffolds). Scaffolds with Trp were chosen due to strong absorbance at 280 nm. While a much smaller sample number would have been sufficient to determine average yield and variability, we chose to determine the yields of all 45 scaffolds containing a Trp due to the simplicity of the assay.<br>Determination of yields for scaffolds of Library 2: 96 scaffolds (out of 192 scaffolds); scaffolds with Trp were chosen due to strong absorbance at 280 nm. While a much smaller sample number would have been sufficient to determine average yield and variability, we chose to determine the yields of all 96 scaffolds containing a Trp due to the simplicity of the assay.
Data exclusions	None
Replication	Thrombin inhibitors M1 to M5 in protease inhibition assay: mean values and SDs of three independent measurements; all attempts of replication were successful<br>MDM2 ligands F-M6 to F-M8, F-M10 in FP assay: mean values and SDs of three independent measurements; all attempts of replication were successful<br>MDM2 ligands M6 to M8, M10 measured by SPR: mean values and SDs of three independent measurements; all attempts of replication were successful
Randomization	N/A; randomization was not applied as the parameters determined (synthesis yield, binding affinity) are measured by instruments (absorption spectrometer, fluorescence polarization, surface plasmon resonance, etc.) that are well standardized by reference compounds (controls).
Blinding	N/A; blinding was not applied as the parameters determined (synthesis yield, binding affinity) are measured by instruments (absorption spectrometer, fluorescence polarization, surface plasmon resonance, etc.) and the result cannot easily be biased by the expectation of the experimenter.

Reporting for specific materials, systems and methods

We require information from authors about some types of materials, experimental systems and methods used in many studies. Here, indicate whether each material, system or method listed is relevant to your study. If you are not sure if a list item applies to your research, read the appropriate section before selecting a response.

Materials & experimental systems

n/a	Involved in the study
☒	☐ Antibodies
☒	☐ Eukaryotic cell lines
☒	☐ Palaeontology and archaeology
☒	☐ Animals and other organisms
☒	☐ Human research participants
☒	☐ Clinical data
☒	☐ Dual use research of concern

Methods

n/a	Involved in the study
☒	☐ ChIP-seq
☒	☐ Flow cytometry
☒	☐ MRI-based neuroimaging
